# Editorial: Role of lipid rafts in anti-microbial immune response volume II

**DOI:** 10.3389/fimmu.2022.1043181

**Published:** 2022-10-06

**Authors:** Maria Cristina Gagliardi, Chih-Ho Lai, Kazuhisa Iwabuchi

**Affiliations:** ^1^ Center for Gender Specific Medicine National Institute of Health (ISS), Rome, Italy; ^2^ Graduate Institute of Biomedical Sciences, Department of Microbiology and Immunology, College of Medicine, Chang Gung University, Taoyuan, Taiwan; ^3^ Department of Medical Research, School of Medicine, China Medical University and Hospital, Taichung, Taiwan; ^4^ Department of Nursing, Asia University, Taichung, Taiwan; ^5^ Department of Pediatrics, Molecular Infectious Disease Research Center, Chang Gung Memorial Hospital, Linkou, Taiwan; ^6^ Department of Infection control Nursing, Graduate School of Health Care and Nursing, Juntendo University, Urayasu, Japan; ^7^ Institute for Environmental and Gender-specific Medicine, Graduate School of Medicine, Juntendo University, Urayasu, Japan

**Keywords:** lipid rafts, pathogen recognition receptors, microbial phagocytosis, immune response, infection

Lipid rafts are specialized areas on membranes that are enriched in glycolipids, sphingolipids, and cholesterol. These lipid rafts, together with other membrane molecules, such as adhesion and signal transduction molecules, mediate a variety of cellular functions. Lipid rafts are mainly involved in recruiting and concentrating molecules and receptors involved in cellular signaling, forming a signal transduction platform. Myeloid innate immune cells, such as neutrophils, monocytes, macrophages and dendritic cells, recruit pathogen recognition receptors, including Toll-like and c-type lectin receptors, into lipid rafts, leading to the formation of phagocytic synapses involved in phagocyte innate immunity. Various types of microbial pathogens, such as viruses (SARS-CoV-2, HIV, influenza virus, Epstein-Barr virus, echovirus, and rhinovirus), bacteria (*Pseudomonas aeruginosa*, *Mycobacterium tuberculosis*, and *Shigella flexneri*), fungi (*Candida albicans*) and parasites (*Plasmodium falciparum*, *Toxoplasma gondii*), have been found to employ host cell lipid rafts as surface platforms, enabling them to interact with, bind to and possibly enter host cells.

Kulkarni et al., summarized that the roles of lipid rafts in pathogen-host interactions suggested that major vault protein (MVP), the main constituent of ribonucleoprotein complexes, is enriched in lipid rafts upon infection with vaccinia virus. MVP is widely expressed in many normal tissues and is overexpressed in many multi-drug-resistant cancer cells. MVP was found to be enriched in lipid rafts following infection of human lung epithelial cells with *Pseudomonas aeruginosa*. Binding of the outer-core oligosaccharide of *Pseudomonas aeruginosa* lipopolysaccharides, activating NF-κB signaling, interleukin (IL)-8 secretion and the induction of apoptosis. Bacterial uptake into the lungs of MVP knockout (MVP-/-) mice was found to be 45% of uptake into the lungs of wild-type mice, suggesting that MVP is recruited to lipid rafts upon exposure to pathogens.

*Helicobacter pylori (H. pylori)* is a Gram-negative pathogen that increases the risk of gastric cancer. *H. pylori* exploits lipid rafts to infect host cells. This infection triggers the clustering of Lewis x antigen (Le^x^) and integrins into lipid rafts, facilitating the adherence of *H. pylori* to the gastric epithelium. Do et al., demonstrate that the cell-free supernatant derived from cells infected with *Lactobacillus rhamnosus* JB3 (LR-JB3) at a multiplicity of infection of 25 had the ability to attenuate the pathogenicity of *H. pylori*. Porcine gastric mucin dramatically up-regulated the expression of *H. pylori* virulence genes, as well as the association between *H. pylori* and IL-8 levels in infected-AGS cells. These findings indicated that LR-JB3 can alter *H. pylori* infection by mediating host cell formation of lipid rafts. The as yet unidentified molecules secreted by LR-JB3 cells are valuable not only for treating *H. pylori* infection, but for diseases mediated by lipid raft signaling, such as cancer, aging, and neurodegenerative diseases.

Lai et al. demonstrate that the gut commensal bacterium *Parabacteroides goldsteinii (P*. *goldsteinii*) MTS01 has been shown to alter gut microbiota composition and reduce cholesterol concentration to mitigate *H. pylori*-induced pathogenesis. *H. pylori* possesses two major virulence factors, vacuolating cytotoxin A (VacA) and cytotoxin-associated gene A (CagA), both of which are involved in its pathogenesis. Probiotics have recently been used to eradicate *H. pylori* infection and reduce the adverse effects of antibiotic-based therapies. *P. goldsteinii* MTS01 is a novel next-generation probiotic with activities that can alleviate specific diseases by altering the gut microbiota. Administration of *P. goldsteinii* MTS01 to *H. pylori*-infected mice was found to alter the composition of the gut microbiota and to significantly reduce serum cholesterol levels, thereby mitigating *H. pylori*-induced gastric inflammation. In addition, the pathogenic effects of *H. pylori* VacA and CagA on gastric epithelial cells were markedly abrogated by treatment with *P. goldsteinii* MTS01. These results indicate that *P. goldsteinii* MTS01 can modulate gut microbiota composition and has anti-virulence activities, suggesting that *P. goldsteinii* MTS01 may be a novel functional probiotic for reducing *H. pylori*-induced pathogenesis.

Severe acute respiratory syndrome coronavirus-2 (SARS-CoV-2), the agent responsible for coronavirus disease 2019 (COVID-19), has had serious deleterious effects on human health worldwide. SARS-CoV-2 has been found to alter host lipid homeostasis. Although lipid rafts have multiple functions in viral replication, their role in SARS-CoV-2 infection remains unclear. Palacios-Rápalo et al., discuss the novel evidence that the cholesterol-rich lipid rafts can act as a platform for SARS-CoV-2 entry, with host cell receptors such as angiotensin-converting enzyme-2, heparan sulfate proteoglycans (HSPGs), human Toll-like receptors, transmembrane serine proteases, CD-147 and HDL-scavenger receptor B type 1 being recruited for their interaction with the viral spike protein. Drugs approved by the U.S. Food and Drug Administration, such as statins, metformin, hydroxychloroquine, and cyclodextrins (methyl-β-cyclodextrin), can disrupt cholesterol-rich lipid rafts to regulate key molecules in the immune signaling pathways triggered by SARS-CoV-2 infection.

Influenza A virus (IAV) is widely disseminated across different species and can cause recurrent epidemics and severe pandemics in humans. IAV particles primarily bind to receptors located in lipid rafts. Statins are a class of drugs commonly used to inhibit cholesterol synthesis, thereby preventing the development and/or progression of cardiovascular diseases. Several studies have investigated whether statins can also block IAV infection and propagation, as well as modulate host immune responses to IAV. Although statins may prevent IAV infections and modulate host immune responses by mitigating cytokine storms, further studies are warranted.

This second issue provides updated research information on the functions of lipid rafts in microbial pathogenesis. Understanding the interactions between pathogens and lipid rafts may provide crucial insights into the mechanisms associated with infectious diseases and aid in the development of novel therapeutic strategies to prevent the diseases caused by these pathogens.

## Author contributions

All authors listed have made a substantial, direct, and intellectual contribution to the work and approved it for publication.

## Conflict of interest

The authors declare that the research was conducted in the absence of any commercial or financial relationships that could be construed as a potential conflict of interest.

## Publisher’s note

All claims expressed in this article are solely those of the authors and do not necessarily represent those of their affiliated organizations, or those of the publisher, the editors and the reviewers. Any product that may be evaluated in this article, or claim that may be made by its manufacturer, is not guaranteed or endorsed by the publisher.

